# The presence of tumor associated macrophages in tumor stroma as a prognostic marker for breast cancer patients

**DOI:** 10.1186/1471-2407-12-306

**Published:** 2012-07-23

**Authors:** Catharina Medrek, Fredrik Pontén, Karin Jirström, Karin Leandersson

**Affiliations:** 1 Center for Molecular Pathology, Jan Waldenströmsgata 59, Skåne University Hospital, Lund University, 20502, Malmö, Sweden; 2 Department of Genetics and Pathology, Rudbeck Laboratory, Uppsala University, 751 85, Uppsala, Sweden; 3 Department of Clinical Sciences, Pathology, Skåne University Hospital, Lund University, 221 85, Lund, Sweden

**Keywords:** Tumor associated macrophages, Tumor stroma, CD163, CD68

## Abstract

**Background:**

Tumor associated macrophages (TAMs) are alternatively activated macrophages that enhance tumor progression by promoting tumor cell invasion, migration and angiogenesis. TAMs have an anti-inflammatory function resembling M2 macrophages. CD163 is regarded as a highly specific monocyte/macrophage marker for M2 macrophages. In this study we evaluated the specificity of using the M2 macrophage marker CD163 as a TAM marker and compared its prognostic value with the more frequently used pan-macrophage marker CD68. We also analyzed the prognostic value of the localization of CD163^+^ and CD68^+^ myeloid cells in human breast cancer.

**Methods:**

The extent of infiltrating CD163^+^ or CD68^+^ myeloid cells in tumor nest versus tumor stroma was evaluated by immunohistochemistry in tissue microarrays with tumors from 144 breast cancer cases. Spearman’s Rho and χ^2^ tests were used to examine the correlations between CD163^+^ or CD68^+^ myeloid cells and clinicopathological parameters. Kaplan Meier analysis and Cox proportional hazards modeling were used to assess the impact of CD163^+^ and CD68^+^ myeloid cells in tumor stroma and tumor nest, respectively, on recurrence free survival, breast cancer specific and overall survival.

**Results:**

We found that infiltration of CD163^+^ and CD68^+^ macrophages into tumor stroma, but not into tumor nest, were of clinical relevance. CD163^+^ macrophages in tumor stroma positively correlated with higher grade, larger tumor size, Ki67 positivity, estrogen receptor negativity, progesterone receptor negativity, triple-negative/basal-like breast cancer and inversely correlated with luminal A breast cancer. Some CD163^+^ areas lacked CD68 expression, suggesting that CD163 could be used as a general anti-inflammatory myeloid marker with prognostic impact. CD68^+^ macrophages in tumor stroma positively correlated to tumor size and inversely correlated to luminal A breast cancer. More importantly, CD68 in tumor stroma was an independent prognostic factor for reduced breast cancer specific survival.

**Conclusion:**

These findings highlight the importance of analyzing the localization rather than merely the presence of TAMs as a prognostic marker for breast cancer patients.

## Background

A malignant tumor is comprised of cancer cells and the tumor microenvironment. The tumor microenvironment consists of extracellular matrix, endothelial cells, fibroblasts and leukocytes, all of which influence the fate of cancer cells and hence the clinical outcome. Tumor associated macrophages (TAMs) have been shown to enhance tumor progression by promoting tumor invasion, migration and angiogenesis [1]. TAMs, which are often abundantly present in malignant tumors, share many common features with the alternatively activated anti-inflammatory macrophages (M2) [2].

Macrophages can be differentiated into either pro-inflammatory M1 macrophages or anti-inflammatory M2 macrophages. M1 macrophages activate type 1 helper T cells (Th1), have the ability to kill pathogens and are tumoricidal. M2 macrophages on the other hand are involved in wound healing where they downregulate the inflammatory reactions, promote angiogenesis, recruit fibroblasts and regulate connective tissue remodeling. Furthermore, M2 macrophages have a weak tumoricidal capability [3].

CD163 is a scavenger receptor upregulated by macrophages in an anti-inflammatory environment [4] and regarded as a highly specific monocyte/macrophage marker for M2 macrophages [5-7].

In 2002 Bingle et al. reported that the majority of publications on TAMs in cancer (including breast cancer) reported a correlation between high TAM infiltration and poor patient outcome [8-11]. All papers in the metaanalysis except one (which used CD31 and thymidine phosphorylase) used CD68 as a marker for TAMs [8]. However CD68, unlike CD163, recognizes both M1 and M2 macrophages [12]. To our knowledge CD163 has not been evaluated as a TAM marker in primary breast cancer. Therefore, the aim of this study was to analyze whether CD163 can be used as a marker for TAMs and to compare its prognostic value with the more frequently used pan-macrophage marker CD68 in a tissue microarray (TMA) with tumors from 144 breast cancer patients.

In melanoma, the presence of TAMs in the tumor stroma (TS) correlated with poor overall survival (OS) [13], while contrasting data have been reported for colorectal cancer [14]. We therefore aimed to examine the importance of localization of TAMs on tumor-promoting capabilities and whether localization in the TS or tumor nest (TN) is of prognostic relevance in breast cancer.

Here we show that infiltration of CD163^+^ and CD68^+^ macrophages into TS, but not TN, is of clinical relevance for breast cancer patients. This highlights the importance of analyzing the localization rather than merely the presence of TAMs as a prognostic marker. While the presence of CD163^+^ macrophages in TS was more strongly associated with less favorable clinicopathological features, CD68^+^ macrophages in TS was a significant independent risk factor for a reduced breast cancer specific survival.

## Methods

### Breast cancer patients

The breast cancer cohort analyzed consists of 144 patients diagnosed with invasive breast cancer at Skåne University Hospital, Malmö, Sweden, between 2001 and 2002. The cohort and TMA construction have previously been described in detail [15-17]. The majority, 109, of the patients had luminal A breast cancer (79%), while 15 patients (11%) had triple-negative/basal-like breast cancer. Immunohistochemical staining of ER, PR, and human epidermal growth factor receptor (HER) was performed as previously described [18] and used as surrogate markers for molecular subtypes [19]. Mean age was 65 years (range 34-97). During follow-up, 41 patients (28%) died and 29 patients (20%) had recurrence. Median follow-up was 6.55 years (range 0.33-7.55 years) for the full cohort and 6.74 years (range 4.88-7.55) for patients alive. Ninety-six patients (67%) had endocrine therapy: of whom 3 patients (2%) had been given aromatase inhibitors (AI) only, 67 (47%) tamoxifen only and 25 patients (17%) both AI and tamoxifen, and in 1 (1%) case unspecified. Of the 30 patients (21%) receiving chemotherapy, 26 (87%) had a combined treatment with fluorouracil, epirubicin and cyclophosphamide (FEC). Ethical approval for the use of breast cancer specimens for this study was obtained from the Ethics Committee at Lund University (ref no 447-07), whereby written consent was not required and patients were offered the option to opt out.

### Immunohistochemistry

Four μm thick TMA sections were mounted onto glass slides and deparaffinised followed by antigen retrieval using the PT-link system (DAKO, Glostrup, Denmark) and then stained in an Autostainer Plus (DAKO) with the EnVisionFlex High pH-kit (DAKO). Primary antibodies included: anti-CD163 (10D6 dilution 1:250; Novocastra), anti-CD68 (dilution 1:1500; DAKO) and anti-DC-LAMP (also known as CD208; a marker for mature myeloid dendritic cells; clone 101E1.01 dilution 1:1000; Dendritics). Immunohistochemistry (IHC) had previously been performed on sections from the same TMA blocks with the anti-Granulin antibody HPA028747 (expressed in tumor-resident bone marrow derived cells responsible for instigation; 1:100; AtlasAntibodies) and the outcome of immunoreactivity was assessed by image analysis [15]. The estimated fraction of cells with nuclear Ki67 expression was denoted as low (0-10%), intermediate (11-25%) and high (>25%) according to previous studies [20]. ER-negativity and PR-negativity was defined as <10% positively staining nuclei, according to current clinical guidelines in Sweden. The CD163 and CD68 staining was scored as the infiltration density of CD163^+^ and CD68^+^ cells with a monocyte/macrophage morphology, ranging from 0 (absent) up to 3 (dense). For statistical analyses, these categories were dichotomized into absent/sparse (0-2) or dense (3) macrophage infiltration. The CD163^+^ and CD68^+^ macrophages were scored in the TS and TN separately. The amount of TS content was scored into the categories ≤50% or >50% of the tumor core as visualized by HE-staining and microscopic evaluation. The CD208/DC-LAMP staining was scored as the presence of CD208^+^ cells with a DC morphology ranging from 0 (absent) – 1 (present). The CD208^+^ cells were present in 25% of all cases (31 out of 121) and were located in the peri-tumoral T cell rich areas only.

### Gene expression analysis

The gene expression levels of CD163 and CD68 in different breast cancer patient groups were analyzed using microarray profile sets, [GenBank:GDS1329] [21] and [GenBank:GDS806] [22], from NCBI Gene Expression Omnibus profiles [23].

### Statistics

Spearman’s Rho and χ^2^ tests were used for comparison of CD163 and CD68 expression and patient and tumor characteristics. Kaplan-Meier analysis and log rank tests were used to illustrate differences in recurrence free survival (RFS), breast cancer specific survival (BCSS) and overall survival (OS) according to CD163 and CD68 expression. Cox regression proportional hazards models were used for estimation of hazard ratios (HR) for death from breast cancer or overall causes according to CD163 and CD68 expression in both uni- and multivariable analysis. Covariates with a *P* value ≤ .05 in the univariable analysis were included in the multivariable analysis. All statistical tests were two sided and *P* ≤ .05 was considered significant. Calculations were performed with IBM SPSS Statistics version 19.0 (SPSS Inc).

For the gene expression data, the means, standard deviations and standard error of the means were analyzed and plotted with GraphPad Prism software. Statistical analyses were carried out with unpaired Student’s *t*-test.

## Results

### Characterization of CD163^+^ and CD68^+^ macrophages in primary breast cancer

**Figure 1:**
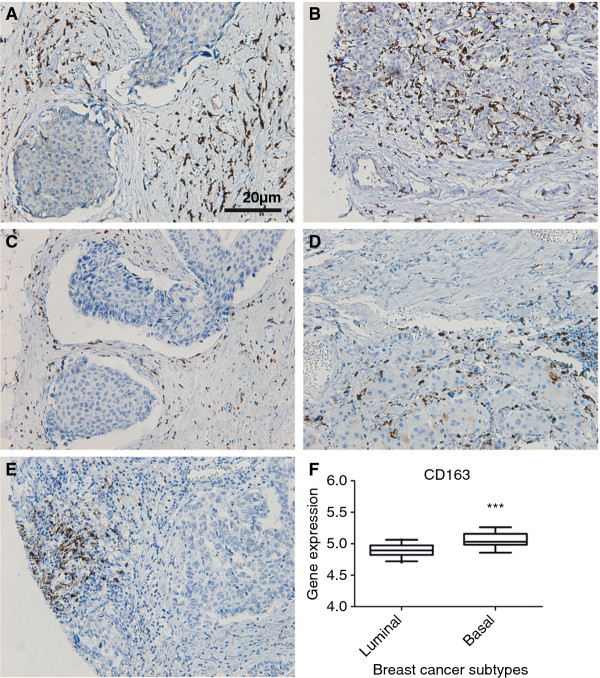
** IHC staining of primary breast tumors.** CD163^+^**AB** and CD68^+^**CD**, cells with a macrophage-morphology were present in both TS and TN. CD208^+^ cells were located in peri-tumoral T cell zones only, **E**. Gene expression levels of CD163 in luminal and basal-like breast cancer, using the microarray profile set [GenBank:GDS1329] [21] from NCBI Gene Expression Omnibus profiles, **F**. *** *P*<0.001.

Due to loss of material or low tumor cell content, 121 (84%) samples were annotated for CD163 in TS, 105 (73%) for CD163 in TN and 108 (75%) samples were scored for CD68 in both TS and TN. As shown in Figure 1A-D, CD163^+^ and CD68^+^ cells with a macrophage-morphology were present in both TS and TN of primary breast cancers. There was a strong correlation between CD163 and CD68 in both TS (*P*<.001) and TN (*P*<.001). There was no correlation between the infiltration density of macrophages in TS and the infiltration density of macrophages in the TN. However, CD163 in TN correlated to the presence of CD208^+^ cells in peri-tumoral T cell zones (*P* = .009). CD208 (DC-LAMP) is a marker for mature myeloid dendritic cells (MDC), which could be reasoned to express CD163 [24]. CD208^+^ cells were only found in the T cell rich peri-tumoral areas and not in TN (Figure 1E).

### Distribution of CD163^+^ and CD68^+^ macrophages in primary breast cancer

To analyze if the localization of CD163^+^ and CD68^+^ macrophages had any correlation to clinical characteristics, the distribution of CD163^+^ and CD68^+^ macrophages in TS and TN was evaluated separately (Table 1 and see Additional file 1). The staining categories initially scored from 0 to 3 in macrophage infiltration density were further dichotomized into two groups with absent/sparse (0-2) or dense (3) macrophage infiltration. Seventeen percent of the tumors were denoted as having dense infiltration of CD163^+^ macrophages in TS, while 9% had dense infiltration in TN. Nine percent had dense infiltration of CD68^+^ macrophages in TS, while 6% had dense infiltration in TN. Hence, the majority of tumors had absent or sparse macrophage infiltration.

**Table 1:** Correlations between CD163 and CD68 in Tumor Stroma and Tumor Nest and clinicopathologic features in primary breast cancer

**Clinicopathologic features**	**CD163**						**CD68**					
	**Tumor Stroma**			**Tumor Nest**			**Tumor Stroma**			**Tumor Nest**		
	**Correlation coefficient**	***P*value (2-tailed)**	**N**	**Correlation coefficient**	***P*value (2-tailed)**	**N**	**Correlation coefficient**	***P*value (2-tailed)**	**N**	**Correlation coefficient**	***P*value (2-tailed)**	**N**
Age	−0.011	.91	121	0.027	.79	105	0.227*	.02	108	−0.114	.24	108
Nodal stage	0.094	.33	108	−0.163	.12	95	0.18	.08	98	−0.008	.94	97
Tumor size	0.339**	.000	121	0.031	.75	105	0.250**	.009	108	0.033	.74	108
Ki67	0.263**	.007	104	0.127	.23	91	0.131	.21	92	0.05	.65	92
NHG	0.326**	.000	121	0.091	.35	105	0.272**	.004	108	0.024	.81	108
Her 2	−0.024	.80	117	−0.077	.44	103	0.079	.42	104	−0.06	.54	104
ER status	−0.422**	.000	121	−0.069	.48	105	−0.047	.63	108	0.11	.26	108
PR status	−0.395**	.000	121	0.066	.50	105	−0.045	.64	108	0.106	.27	108
Basal	0.621**	.000	118	−0.014	.89	103	0.173	.08	104	−0.102	.31	104
Triple negative	0.624**	.000	118	−0.014	.89	103	0.173	.08	104	−0.102	.31	104
Luminal A	−0.514**	.000	114	0.076	.45	100	−0.237*	.02	100	0.141	.16	100
Luminal B	0.034	.72	116	−0.054	.59	102	0.138	.17	102	−0.047	.64	102

### Distribution of CD163^+^ and CD68^+^ macrophages in luminal a and triple-negative/basal-like breast cancer

CD163^*+*^ and CD68^*+*^ macrophages were more equally distributed among the patients with luminal A breast cancer (see Additional file 1). The majority had absent/sparse macrophage infiltration density in both the TS and TN. Dense infiltration of CD163^+^ and CD68^+^ macrophages in TS was observed in only 8% and 6% of the cases respectively. Eighty percent of the triple-negative/basal-like breast cancer patient had dense infiltration of CD163^*+*^ macrophages in TS, while 23% had dense infiltration of CD68^*+*^ macrophages in TS. This was not due to an increase in the amount of TS since we saw an inverse correlation between the amount of TS and the density of TS-associated CD163^+^ and CD68^+^ TAMs (see Additional file 2). We did not find any correlation between the amount of TS and breast cancer subtypes in our cohort (data not shown). The majority of the cases (92% for CD163^+^ and 100% for CD68^+^) had absent/sparse macrophage infiltration in TN.

### Correlations between CD163^+^ and CD68^+^ macrophages and clinicopathological characteristics

Breast cancer tumors with dense infiltration of CD163^+^ macrophages in the TS were of higher grade (*P*<.001), larger size (*P*<.001) and had a higher proliferation index as indicated by Ki67 positivity (*P* = .007). Dense infiltration of CD163^+^ macrophages in the TS was further associated with estrogen receptor (ER) negativity (*P* = .001), progesterone receptor (PR) negativity (*P*<.001), triple-negative/basal-like breast cancer (*P*<.001), inversely correlated with luminal A breast cancer (*P*<.001) (Table 1) and correlated with the extent of granulin (GRN) expression (*P* = .01) (see Additional file 3). CD163^+^ macrophages in TN did not correlate with any clinicopathological features.

To further evaluate the association between CD163^+^ macrophages and different breast cancer subtypes, we analyzed the gene expression levels of CD163 in both basal-like and luminal breast cancer, using a publically available gene expression array dataset [GenBank:GDS1329] [21] from NCBI Gene Expression Omnibus profiles [23]. In line with the findings from the TMA-based analysis, basal-like breast cancer had significantly higher gene expression levels of CD163 (*P*<.001) compared to luminal breast cancer (Figure 1F), but also of CD68 (*P*<.05) (data not shown).

Dense infiltration of CD68^+^ macrophages in the TS positively correlated with large tumor size and high grade and inversely correlated with luminal A breast cancer. There was no significant association between CD68^+^ macrophages in TN and any clinicopathological features.

### Impact of CD163^+^ and CD68^+^ macrophages on survival

**Figure 2:**
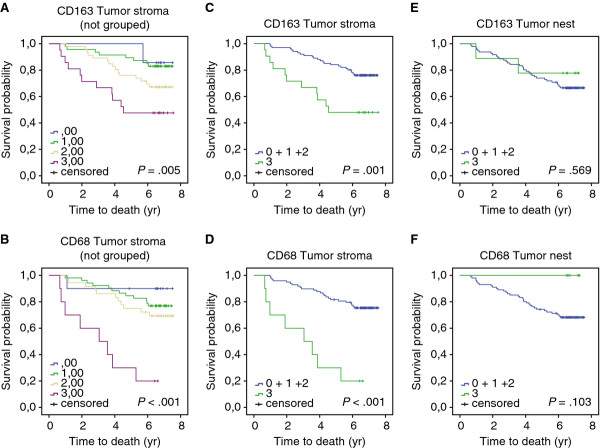
** OS according to the infiltration density of CD163**^**+**^**and CD68**^**+**^**cells in primary breast cancer.** OS curves according to the infiltration density of CD163^+^ and CD68^+^ cells in TS. Non grouped data **A**,**B**. Grouped data **C**,**D**,**E**,**F**. OS curves according to the infiltration density of CD163^+^ and CD68^+^ cells in TS *C,D* and TN *E,F*.

**Figure 3:**
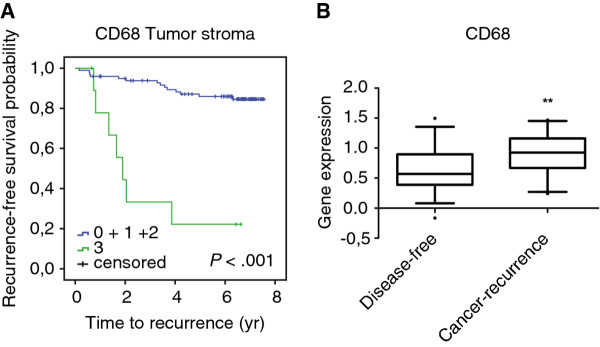
** RFS according to the infiltration of CD68**^**+**^**cells in TS.** RFS curve according to the infiltration density of CD68^+^ cells in TS, **A**. Gene expression levels of CD68 in breast cancer patients under endocrine therapy, who were disease-free or who had recurrence, using the microarray profile set [GenBank:GDS806] [22] from NCBI Gene Expression Omnibus profiles **B**. ** *P*<0.01.

We found that dense infiltration of CD163^+^ and in particular CD68^+^ macrophages in TS correlated with poor OS (Figure 2A-D and Table 2) and poor BCSS (Table 2). CD68^+^ macrophages in TS further correlated to recurrence (Figure 3A and Table 2). Using a publically available gene expression array dataset [GenBank:GDS806] [22] from NCBI Gene Expression Omnibus profiles [23] we were able to identify that breast cancer patients receiving endocrine therapy, who had recurrence, had significantly higher gene expression levels of CD68 (Figure 3B).

**Table 2:** Univariate Cox Regression Analyses for OS, BCSS and RFS

**Variables**	**OS**			**BCSS**			**RFS**		
	**HR**	**95% CI**	***P***	**HR**	**95% CI**	***P***	**HR**	**95% CI**	***P***
Age	1.07	1.04-1.09	.000	1.05	1.02-1.09	.003	1.04	1.01-1.07	.012
Lymph node status									
- vs. +	2.59	1.30-5.14	.007	5.57	1.85-16.78	.002	6.70	2.52-17.80	.000
Tumor size									
>20 vs. ≤20 mm	2.53	1.31-4.89	.006	3.04	1.19-7.78	.02	3.14	1.34-7.10	.006
Ki67	3.00	1.51-5.96	.002	3.99	1.38-11.50	.01	1.68	0.82-3.44	.16
Grade	2.11	1.28-3.49	.004	5.14	2.03-13.00	.001	3.18	1.62-6.25	.001
HER	3.75	1-57-8.99	.003	7.69	2.98-19.86	.000	7.13	3.01-16.90	.000
ER	0.39	0.19-0.79	.009	0.23	0.10-0.55	.001	0.28	0.13-0.60	.001
CD163 in TS	2.66	1.28-5.55	.009	3.20	1.20-8.54	.02	2.12	0.83-5.39	.12
CD68 in TS	0.16	0.07-0.35	.000	15.2	5.45-42.45	.000	9.24	3.64-23.48	.000
CD163 in TN	0.32	0.04-2.37	.27	1.45	0.33-6.32	.62	1.06	0.25-4.55	.94
CD68 in TN	0.05	0.00-21.70	.33	0.44	0.00-169.12	.46	0.04	0.00-43.30	.37

Abbreviations: *OS*, overall survival; *BCSS*, breast cancer specific survival; *RFS*, recurrence free survival; *HR,* hazard ratio.

There was no observed correlation between CD163^+^ or CD68^+^ macrophages in TN with OS, BCSS or RFS (Table 2).

**Figure 4:**
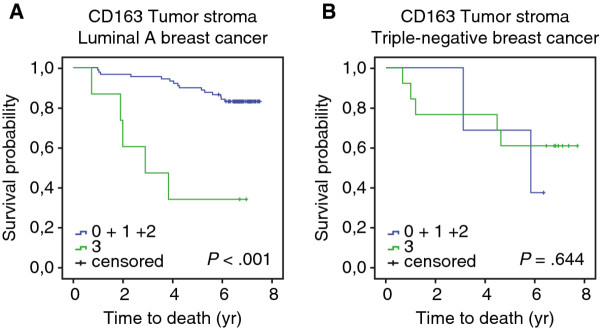
** Infiltration of CD163**^**+**^**cells in different breast cancer subtypes.** OS curves according to the infiltration density of CD163^+^ cells in TS, for patients with luminal A breast cancer **A**, or triple-negative/basal-like breast cancer **B**.

Since CD163^+^ macrophages in the TS positively correlated with triple-negative/basal-like breast cancer and inversely correlated with luminal A breast cancer (Table 1 and 2), the prognostic value of CD163^+^ macrophages in TS was evaluated separately in the two different breast cancer subtypes (Figure 4A, B). Luminal A breast cancer patients with dense infiltration of CD163^+^ macrophages had a worse OS (Figure 4A). The majority (74%) of the luminal A breast cancer patients received endocrine therapy.

There was no difference in patient outcome according to CD163^+^ macrophage infiltration in TS in the triple-negative/basal-like breast cancer patient group (Figure 4B).

Multivariate analyses revealed that CD163 was not an independent risk factor for OS, BCSS or RFS (Table 3). Dense infiltration of CD68^+^ macrophages in TS was not an independent risk factor for OS and RFS but was an independent risk factor for BCSS (HR = 0.12; 95% CI, 0.02 to 0.72; *P* = .02), (Table 3).

**Table 3:** Multivariate Cox Regression Analyses for OS, BCSS and RFS

**Variables**	**OS**			**BCSS**			**RFS**		
	**HR**	**95% CI**	***P***	**HR**	**95% CI**	***P***	**HR**	**95% CI**	***P***
Age	1.07	1.03-1.10	.000	1.05	1.00-1.09	.03	1.03	1.00-1.07	.07
Lymph node status									
- vs. +	1.81	0.87-3.82	.12	3.28	1.01-10.63	.05	4.94	1.77-13.80	.002
Tumor size									
>20 vs. ≤20 mm	1.58	0.65-3.81	.31	2.47	0.60-10.08	.21	2.12	0.72-6.24	.17
Ki67	3.06	1.05-8.93	.04	4.40	0.67-28.82	.12	0.45	0.13-1.53	.20
Grade	0.63	0.27-1.49	.29	0.86	0.20-3.67	.84	2.25	0.78-6.56	.14
HER	3.45	1.16-10.24	.03	5.37	1.61-17.90	.006	4.46	1.63-12.20	.004
ER	0.47	0.17-1.35	.16	0.39	0.11-1.41	.15	0.37	0.12-1.13	.08
CD163 in TS	0.62	0.24-1.58	.32	0.74	0.23-2.38	.61	0.91	0.30-2.72	.86
CD68 in TS	2.96	0.87-10.11	.08	0.12	0.02-0.72	.02	0.29	0.08-1.10	.07

Abbreviations: *OS*, overall survival; *BCSS,* breast cancer specific survival; *RFS*, recurrence free survival; *HR*, hazard ratio.

## Discussion

CD68 is a pan-macrophage marker frequently used as a marker for TAMs. However, CD68 recognizes both tumoricidal M1 and anti-inflammatory M2 macrophages. This may explain previous contradictory data showing that dense infiltration of TAMs was correlated to both good and poor patient outcome [8]. CD163 is a highly specific monocyte/macrophage marker that has recently been suggested to be expressed primarily by the anti-inflammatory subtypes of macrophages [4-7,25]. In this study we aimed to compare CD163 with the more frequently used pan-macrophage marker CD68. In addition we analyzed if the localization of macrophages in primary breast cancer could be of clinical relevance.

In our patient cohort, infiltration of TAMs in the TN was not correlated with any clinicopathological features and did not relate to OS, BCSS or RFS. The involvement of TN located TAMs for patient outcome should not however be fully excluded. In melanoma, for instance CD68^+^ TAMs in the TN positively correlated with both poor OS and RFS, however it did not fall out as an independent risk factor upon multivariate analysis [13]. In endometrial cancer and gastric cancer, dense infiltration of CD68^+^ cells in the TN positively correlated with fewer recurrences [26,27] and hence suggested a beneficial effect of TAMs in the TN.

In our study, dense infiltration of CD163^+^ macrophages located in TS correlated with grade, tumor size, subtypes and receptor status. In line with previous papers reporting that the content of TAMs inversely correlates with ER expression in breast cancer [28,29], CD163^+^ macrophages located in TS correlated with ER negativity. Furthermore, CD163^+^ macrophages in TS correlated with triple-negative/basal-like breast cancer and inversely with luminal A breast cancer. It has previously been suggested that the amount of TS correlated with a worse prognosis in triple-negative/basal-like breast cancer [30]. In our cohort we did not find a correlation between the amount of TS and breast cancer subtypes, but we found an inverse correlation between the amount of TS and TS-associated CD163^+^ and CD68^+^ TAMs, rather strengthening the relevance of our findings. Further investigation is needed to understand what particular factors regulate the recruitment and activation of TAMs in the different tumor compartments.

Elkabets et al. recently reported that human tumors can attract GRN expressing hematopoietic cells, which promote malignancy by activating stromal tissue fibroblasts [15]. These cells were located in close proximity to fibroblasts within the TS in mice. Notably, using the same breast cancer TMA as in this study, GRN expression was found to correlate with the same clinicopathological features as CD163^+^ macrophages in TS [15] as well as with the density of CD163^+^ macrophages in TS. This suggests that the CD163^+^ macrophages located in TS could represent GRN^+^ hematopoietic cells.

Triple-negative/basal-like and luminal A breast cancers had different recruitment or differentiation patterns of CD163^+^ and CD68^+^ macrophages in TS. According to our data, triple-negative/basal-like breast cancers seem to harbor more CD163^+^ than CD68^+^ cells within the TS. One explanation for the difference between the presence of CD163^+^ and CD68^+^ macrophages in TS among triple-negative/basal-like breast cancer patients could be that CD68 is less expressed on mature M2 macrophages or monocytes. Another explanation could be that CD163 identifies another subset of cells. Although CD163 has been shown to be a specific monocyte/macrophage marker for IHC studies, flow cytometric analyses have shown that MDCs express CD163 [24]. We can exclude that the majority of the CD163^+^ cells are MDCs since the CD208^+^ mature MDCs in this breast cancer cohort were only located in the peri-tumoral T cell rich areas. However, it cannot be fully excluded that there might be CD163^+^ immature myeloid derived cells among the CD163^+^ cells in TS. These CD163^+^ immature myeloid derived cells could be myeloid derived suppressor cells which have been shown to enhance tumor progression by having an immunosuppressive effect on anti-tumor effectors [31].

Although CD163^+^ macrophages in TS correlated with triple-negative/basal-like breast cancer it did not confer a prognostic value in this group. This could be due to the small size of the group and their worse overall prognosis, making it difficult to detect any significant differences in survival. However, it had a prognostic value for luminal A breast cancer patients.

Interestingly, CD68^+^ macrophages located in TS positively correlated with grade, tumor size and inversely correlated with luminal A breast cancer. It further correlated with poor OS and RFS in the univariate analysis. More importantly, high density of CD68^+^ macrophages in TS was an independent predictor of reduced BCSS. Even though CD163 and CD68 are expressed on the same cells and with strong correlations with each other, our data indicate that they also define immune subpopulations, which affect patient outcome differently and may therefore have distinct immune functions. In TS, the broader pan-macrophage marker CD68 had a better prognostic impact on BCSS compared to the anti-inflammatory CD163 marker, which is surprising since CD163 also seems to stain cells of myeloid origin other than TAMs. CD163^+^ cells were in particular abundant in triple-negative/basal-like breast cancer, which was a small patient group in our TMA. It would therefore be interesting to see if CD163 would have a prognostic value in a larger triple-negative/basal-like cohort.

## Conclusion

Taken together, infiltration of CD163^+^ and CD68^+^ macrophages into TS, but not TN, is of clinical relevance for breast cancer patients and highlights the importance of analyzing the localization rather than merely the presence of TAMs as a prognostic marker. While the presence of CD163^+^ macrophages in TS was more strongly associated with less favorable clinicopathological features, CD68^+^ macrophages in TS was a highly significant independent risk factor for a reduced BCSS.

## Abbreviations

TAMs, Tumor Associated Macrophages; Th1, Type 1 Helper T cells; MDC, Myeloid Dendritic Cells; IHC, Immunohistochemistry; TMA, Tissue Microarray; TS, Tumor Stroma; TN, Tumor Nest; OS, Overall Survival; BCSS, Breast Cancer Specific Survival; RFS, Recurrence Free Survival; HR, Hazard Ratios; NHG, Nottingham Histological Grade; ER, Estrogen Receptor; PR, Progesterone Receptor; HER, Human Epidermal growth factor Receptor; GRN, Granulin; AI, Aromatase Inhibitors; FEC, Fluorouracil Epirubicin and Cyclophosphamide.

## Competing interests

The authors declare that they have no competing interests.

## Authors’ contributions

All authors made substantial contributions to the conception and design of the study, acquisition of data, analysis and interpretation of the data. Further they were involved in drafting the manuscript or revising it. All authors read and approved the final manuscript.

## Pre-publication history

The pre-publication history for this paper can be accessed here:

http://www.biomedcentral.com/1471-2407/12/306/prepub

## Supplementary Material

Additional file 1: Distribution of CD163+ and CD68+ macrophages in primary breast cancer. Click here for file

Additional file 2: **Correlation between the amount of tumor stroma content and the density of CD163**^+^**and CD68**^+^**TAMS in TS.** Click here for file

Additional file 3: Correlation between CD163 in tumor stroma and granulin expression in primary breast cancer. Click here for file
