# Supplementary material for: The presence of tumor associated macrophages in tumor stroma as a prognostic marker for breast cancer patients
Source: BMC Cancer. 2012 Jul 23;12:306. doi: 10.1186/1471-2407-12-306 (PMC3414782; doi:10.1186/1471-2407-12-306)
Supplement: Additional file 1: — Distribution of CD163+ and CD68+ macrophages in primary breast cancer. [file 1471-2407-12-306-S1.pdf]

**Additional file 1.**	Distribution of CD163+ and CD68+ macrophages in primary breast cancer					
	Whole cohort		Luminal A		Triple-negative/basal-like	
	CD163	CD68	CD163	CD68	CD163	CD68
Dense infiltration in tumor stroma (%)	17	9	8	6	80	23
Dense infiltration in tumor nest (%)	9	6	10	9	8	0
