# Supplementary material for: The presence of tumor associated macrophages in tumor stroma as a prognostic marker for breast cancer patients
Source: BMC Cancer. 2012 Jul 23;12:306. doi: 10.1186/1471-2407-12-306 (PMC3414782; doi:10.1186/1471-2407-12-306)
Supplement: Additional file 2: — Correlation between the amount of tumor stroma content and the density of CD163+and CD68+TAMS in TS. [file 1471-2407-12-306-S2.pdf]

Additional file 2.

Correlation between the amount of tumor stroma content and the density of $CD163^+$ and $CD68^+$ TAMs in TS.

Clinicopathologic features	Tumor stroma content		
	Correlation coefficient	*P* value (2-tailed)	N
CD163 in TS	-0,300**	.001	112
CD68 in TS	-0,145**	.000	102
