# Supplementary material for: The presence of tumor associated macrophages in tumor stroma as a prognostic marker for breast cancer patients
Source: BMC Cancer. 2012 Jul 23;12:306. doi: 10.1186/1471-2407-12-306 (PMC3414782; doi:10.1186/1471-2407-12-306)
Supplement: Additional file 3: — Correlation between CD163 in tumor stroma and granulin expression in primary breast cancer. [file 1471-2407-12-306-S3.pdf]

Additional file 3.

Correlation between CD163 in tumor stroma and granulin expression in primary breast cancer.

Clinicopathologic features	CD163		
	Tumor stroma		
	Correlation coefficient	*P* value (2-tailed)	N
Granulin	0.248**	.01	108
